# Vehicle Motion Alarms: Necessity, Noise Pollution, or Both?

**DOI:** 10.1289/ehp.119-a30

**Published:** 2011-01

**Authors:** David C. Holzman

**Affiliations:** **David C. Holzman** writes on science, medicine, energy, economics, and cars from Lexington and Wellfleet, MA. His work has appeared in *Smithsonian*, *The Atlantic Monthly*, and the *Journal of the National Cancer Institute*

Noise pollution chips away at the public health, interfering with our immune systems, learning, and sleep, boosting stress hormones, and contributing to cardiovascular maladies, even at levels too low to cause hearing damage.^1^ If annoyance level is any indication, backup beepers may be one of the most harmful noises. In a 2010 report titled *Technology for a Quieter America*, the National Academy of Engineering cited backup beepers as one of the six top noise sources people associated with behavioral and emotional consequences.^2^ And although no studies to date have assessed the public health impact of backup beepers, the unpredictability and lack of control over when the sounds are heard are characteristics that normally raise noise’s impact on public health, says Arline Bronzaft, chair of the Noise Committee of the Mayor’s Grow NYC (formerly Council on the Environment).

During Boston’s Big Dig project, which rerouted much of the traffic through the heart of the city, including a major highway, people lodged more complaints about noise than about any other annoyance factor and far more complaints about backup beepers than any other noise source, says Erich Thalheimer, project’s noise control officer and the lead noise engineer at Parsons Brinckerhoff, Boston. Similarly, backup beepers topped another list, with 20 state departments of transportation identifying them as a problem in generating nighttime construction noise.^3^

For all their ubiquity, backup beepers are poorly designed for their job, and some of their most annoying attributes are part of that poor design, says Chantal Laroche, a professor in the Audiology/Speech Language Pathology Department at the University of Ottawa, Canada, who has devoted much of her career to investigating the practical shortcomings of alarm sounds. Their single tones, with a typical volume of 97–112 decibels (dB) at the source, are loud enough to damage hearing^4^ and can be heard blocks from the danger zone, says Thalheimer. Their sound is so commonplace that their warning can lose its authority through the cry-wolf phenomenon.^5^ For reasons having to do with the physics of sound, they also are notoriously hard to localize, further undermining their utility, says Laroche.

Robert Andres, a principal with the consulting group Environmental and Safety Associates and technical advisor for the advocacy group Noise Free America, takes a slightly different view. “I don’t believe that backup beepers are necessarily poorly designed for the job. The ‘job’ is to warn people around machinery and, in most environments they do this well by providing a sound that is unique to the surroundings, loud enough to be heard under a variety of circumstances, relatively directional, and easily understood to be a warning,” he says. “Problems arise when multiple beepers are present at a site or the alarm creates an annoyance beyond the danger zone.”

Technologies that could mitigate the problems with backup beepers have existed for around two decades. Nonetheless, the conventional single-tone backup alarm still dominates roads and construction sites.

Now Congress has passed a bill calling for a new set of motion alarms to protect pedestrians—especially the blind—from being surprised by electric vehicles (EVs) and by those hybrid electric vehicles (HEVs) that can run entirely on electricity and that therefore can be exceptionally quiet at slow speeds.^6^ Will “belling” EVs and HEVs be optimally protective and minimally annoying, or will the mistakes of the past be repeated?

## Uncharacterized Public Health Effects

Although the human health impact of vehicle backup beepers has not been studied, at least one potential surrogate study suggests intrusive beeping sounds are not benign. Margaret Topf, now retired from the University of Colorado, Denver, compared rapid eye movement (REM), a measure of sleep quality, in a control group and in women in a simulated critical care unit (CCU) who listened to an audiorecording of actual hospital nighttime sounds. The control group averaged 83 minutes of REM sleep versus 45 minutes for women in the simulated CCU.^7^ (REM sleep optimally makes up about 20% of total sleep time.^8^)Mean sound levels in CCUs are typically around 55–65 dB, with peaks from hospital equipment beepers exceeding 80 dB.^7^

There may be no proof of harm from backup beeper noise, but there is evidence that beepers do not protect life and limb as well as hoped. An investigation by the federal Occupational Safety and Health Administration (OSHA) found that an original equipment manufacturer backup alarm failed to prevent two-thirds of backover accidents analyzed.^9^ In a vote of no confidence in backup beepers, Washington State established a requirement for a spotter at all times—someone who alerts the driver if a pedestrian steps behind the machinery.^10^ Some 183 fatal backovers are estimated to occur annually, with 44 of those attributed to nonpassenger vehicles, according to the U.S. National Highway Traffic Safety Administration (NHTSA).^11^

Laroche has extensively studied the nature of single-tone beepers and the human response thereto. Twenty years ago, as she was finishing her PhD, her supervisor suggested she turn her attention to noise and safety. She began with a field trip to a construction site where she measured the decibel levels of backup beepers. She says she was surprised to find that sound levels varied by as much as 20 dB over distances as short as 6 inches. The variations were caused by the interactions of sound waves emanating directly from the beepers with those reflecting from surfaces, a well-understood phenomenon that can raise or lower the sound level depending on the phase of the waves at the site of interaction.

Today Laroche says variations in sound intensity and their lack of linear correspondence to distance and direction from the truck speakers broadcasting the sound make it difficult for a human to localize the signal’s origin. Furthermore, backup beepers typically broadcast a frequency of around 1,000 Hz, but the frequencies humans use preferentially to localize sound are those greater than 1,600 Hz and less than 800 Hz, says Judy Edworthy, a professor of applied psychology at the University of Plymouth, UK.

Finally, perception is everything when it comes to alarm sounds. Researchers have found that students working at a cognitive task responded to alarms of differing reliability (25%, 50%, and 75% reliability, respectively) at a rate that largely matched the alarms’ reliability.^5^ That is, in the case of alarms that accurately indicated a true emergency only 25% of the time, the majority of the students tended to respond to them only 25% of the time. Another study suggests backup beepers may be ineffective warnings for very young children.^12^ In this study, the researchers asked 33 preschoolers to walk behind a stationary vehicle twice. During the second time, the backup alarm was engaged. Although half the children hesitated or looked toward the beeping vehicle, none of them responded with avoidance behavior. The authors suspected all the children would have been injured had this been an actual backup situation.

## The Best Beeper for the Job?

More than anything, single-tone backup alarms, with all their faults, are a holdover from the past. They evolved from electromechanical buzzers, which were widely used as warning signals during the first half of the twentieth century because of simplicity and low cost, says Henry Morgan, director and general manager of Brigade Electronics in Dartford, UK. And they do meet the relevant OSHA regulation, 1926.601(b)(4), which specifies that vehicles with obstructed rear views must have either a reverse signal alarm audible above the surrounding noise level or a human spotter.^13^

Thus, the regulations do not preclude alternative technologies that are less annoying and more effective, and which have been practical for as long as two decades, Thalheimer says. These alternatives include backup beepers that manually or automatically adjust their noise output up or down according to ambient noise. At their lowest setting of 95 dB, Thalheimer says these alarms are about three-quarters as loud as standard backup beepers.

Still another alternative is the broadband beeper, a device that has the same cadence as the conventional beeper but broadcasts a “white-noise, whooshing sound,” says Thalheimer, who has no financial interest in or affiliation with Brigade Electronics, the manufacturer. He explains the sound is still readily audible behind the vehicle and is more easily localizable than a single-tone beeper, but the white noise is masked by community noise, so it is much less annoying to the public.

But the regulations don’t allow for the latest alternative technology: backup cameras and radar systems. “We’re hoping OSHA will change this rule to push the use of [these] systems,” says Scott Schneider, director of occupational safety and health at the Laborers’ Health and Safety Fund of North America, a nonprofit joint labor management organization affiliated with the Laborers’ International Union of North America. And in fact, as this article was going to press, Schneider said NHTSA is proposing to require video cameras on all vehicles weighing less than 10,000 lbs—a measure that would include cars, sport utility vehicles, and small trucks.^14^

## Late Adopters

Several reasons explain why conventional nonadaptable backup beepers remain by far the most common reverse alarms. “The company that manufactures and sells a machine with a motion alarm is in a catch-22,” says Andres. “They do not know specifically where the equipment will be used during its life cycle or what the background noise level will be in any given circumstance. Thus, they will often opt for the loudest—and often cheapest—alarm possible.”

To compound the problem, OSHA regulations preclude the equipment owner from modifying the alarm without the manufacturer’s approval. “Although a subsequent interpretation also allows this decision to be made by a qualified engineer, presumably after a risk assessment, there are few willing to make a decision to reduce the sound level or duration of an alarm that could later be construed in our litigious environment as compromising safety,” Andres explains.

“It boils down to cost and complacency of the owner of the vehicle,” says Thalheimer. “If you buy a quarter-million-dollar vehicle, it will come with as cheap a backup alarm as the manufacturer can get away with. [If sued], you can tell the court you did not tamper with the manufacturer’s backup alarm, and it helps get you off the hook in many cases.”

Adding additional inertia, many in the field are unaware of the research that criticizes conventional alarms. Engineer Kerry Cone, immediate past chairman of the Society of Automotive Engineers Sound Level Technical Committee, is skeptical of broadband alarms, because he says that people are “imprinted” on conventional beepers, and he doubts they would respond as readily to a signal that he says sounds more like airbrakes. “You need to understand that we’re dealing with human safety,” he says. He was not familiar with Laroche’s research, but he does say, “We investigate new concepts all the time.”

Some evidence suggests change is afoot. Under the New York City Department of Environmental Protection’s 2007 construction noise regulation, which Thalheimer played a large role in developing, Brigade’s white noise alarms or tonal alarms that can be set to quieter levels are required for after-hours operation and use in sensitive areas such as near schools, hospitals, and homes for the aged.^15^ These revised regulations earned the department and Parsons Brinckerhoff the 2010 Safe-in-Sound Award in the category for Innovation in Hearing Loss Prevention in the Construction Sector, an award presented by the National Institute for Occupational Safety and Health in partnership with the National Hearing Conservation Association.^16^ Thalheimer says he recommends white noise alarms on every job he oversees.

## Belling the Car

While anti-noise advocates work to quiet construction vehicles, some groups want to make EVs and HEVs louder. Like EVs, some HEVs run mostly or entirely on electricity at low speeds and thus are exceptionally quiet. Using statistics from 12 states, the NHTSA showed that HEVs were twice as likely as nonhybrid gasoline-powered vehicles to collide with pedestrians.^17^ The relevant excess crashes all occurred at low speed, such as while exiting driveways or starting up in traffic. The NHTSA points out the incidence rates provided in the study should be interpreted with caution due to the small sample size. Nevertheless, the difference in accident rates was statistically significant.

A movement led by advocates for the blind seeks to require add-on sounds for these quieter vehicles. Although some locations now have signals indicating walk cycles,^18^ in many locations blind people still cross the street by listening for traffic, explains Deborah Kent Stein of the National Federation of the Blind (NFB). Lawrence Rosenblum, a psychology professor at the University of California, Riverside, performed audibility experiments on HEVs at the request of the NFB.^19^ “When hybrids are moving slowly, five miles per hour, they are substantially less audible, and depending on background context, we feel dangerously so,” Rosenblum says.

Nonetheless, Rosenblum says additional sound is needed only in limited venues—parking lots, driveways, and the like—and is not needed at all beyond about 20 mph, when tire and wind noise become unmistakeable. Further, “It’s likely that the added sound needs to be absolutely minimal. You need very little sound to engage the brain,” he says, adding that the add-on sounds could be effective at decibel levels lower than the engine purr of current gas-powered cars.

Some automakers are already implementing add-on sounds. The GM Volt’s add-on resembles a car horn,^20^ while the Nissan Leaf sounds like swoosh.^21^ Several other manufacturers are investigating the possibility of add-on sounds.^22^ Rosenblum believes any add-ons should sound like a quietly running car engine. “You don’t want something that will distract pedestrians from another car that’s approaching; you want something that fits the soundscape.”

The NFB and other advocates pushed for legislation to ensure the use of add-on sounds, provisions that were included in this year’s Motor Vehicle Safety Act.^23^ That bill stalled, but in mid-December 2010 the House and Senate both easily passed versions of the Pedestrian Safety Enhancement Act that, if signed by the President, will require the U.S. Department of Transportation to promulgate standards for add-on sounds within 18 months.^6^

The issue has also gone global. The United Nations is devising an international standard for minimum sound regulations for HEVs and EVs, and Japan has established voluntary regulations calling for minimal levels of sound to be broadcast at speeds of less than 20 kph (12.4 mph).^24^

Anti-noise advocates are wary of add-on sounds. Les Blomberg, executive director of the nonprofit Noise Pollution Clearinghouse, doesn’t buy the need for artificially noisy electric cars, at least not yet. “We have very little data [on the benefits of add-on sounds], and what data we have is ambiguous when you look at the total societal impact,” he says. “Advocates [for add-on sounds] see the vehicles as too quiet, and the solution is to make them louder. I see the problem as the environment is too loud to hear them, and the solution is to quiet the environment. If and when trucks are electric, the streets will be far quieter than they are now.”

And regarding the NHTSA study on HEV collisions,^17^ Blomberg wonders whether the excess of such accidents with HEVs is due to pedestrians’ lack of adjustment to this new phenomenon of quiet cars. He points to alternatives to putting more noise in the environment including pedestrian education, rearview cameras in vehicles, and better parking lot design.

Anti-noise advocates also propose the development of transponders that blind pedestrians could carry around to alert them to the presence of quiet cars. “That’s worth studying,” says Jay Joseph, chairman of the Society of Automotive Engineers Vehicle Sound for Pedestrians Committee. “The blind community feels that they shouldn’t be dependent on some battery-powered device for their overall safety as a pedestrian, which is a fair point. I think in the future there is some potential for those kinds of applications, but they need to be refined and appeal to the population that perceives themselves at risk.”

If past is prologue, however, any add-on sound could remain the status quo for many years. It better be good.

## Figures and Tables

**Figure:**
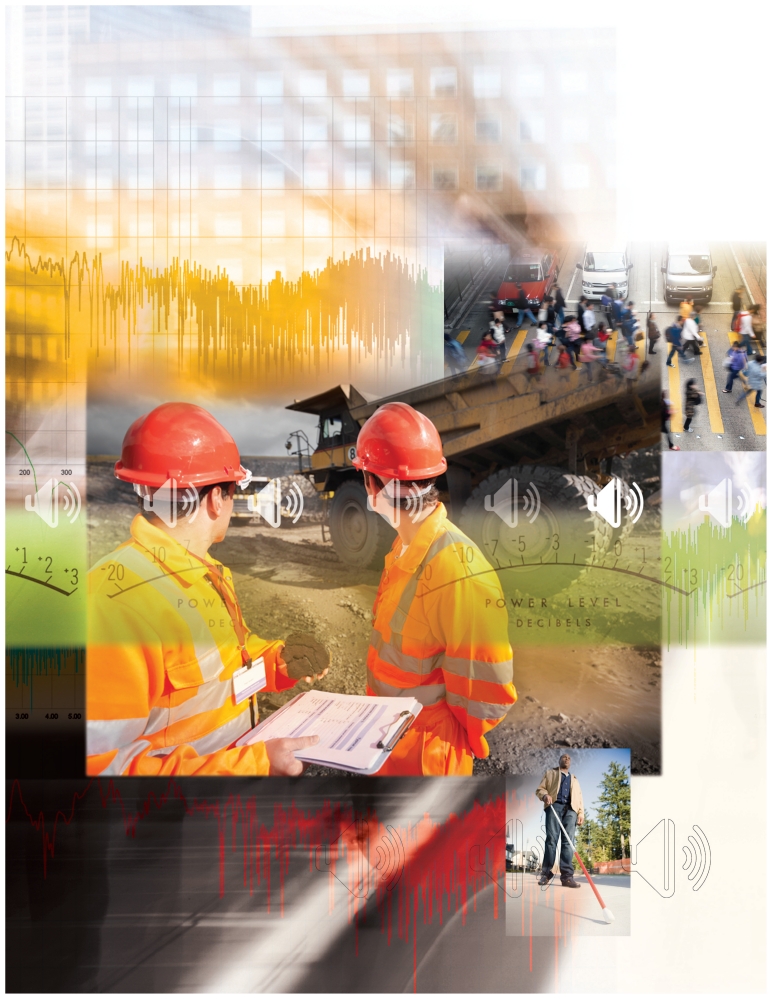


## References

[1] Stansfeld SA, Matheson MP (2003). Noise pollution: non-auditory effects on health. Br Med Bull.

[2] Committee on Technology for a Quieter America (2010). Technology for a Quieter America.

[3] FHWA Effective Noise Control During Nighttime Construction.

[4] NIDCD Sound Ruler, Text-Only Version [website].

[5] Bliss JP (1996). Human probability matching behavior in response to alarms of varying reliability. Ergonomics.

[6] S.841 Pedestrian Safety Enhancement Act of 2010.

[7] Topf M, Davis JE (1993). Critical care unit noise and rapid eye movement (REM) sleep. Heart Lung.

[8] NINDS Brain Basics: Understanding Sleep [website].

[9] Purswell JP, Purswell JL, Bittner AC (2001). The effectiveness of audible backup alarms as indicated by OSHA accident investigation records. Advances in Occupational Ergonomics and Safety.

[10] Schneider S (2009). Preventing backovers in work zones.

[11] NHTSA (2006). Estimation of Backover Fatalities.

[12] Sapien R (2003). Children’s response to a commercial back-up warning device. Inj Prev.

[13] OSHA Safety and Health Regulations for Construction. Motor Vehicles, Mechanized Equipment, and Marine Operations. Motor Vehicles. Standard 1926.601(b)(4).

[14] NHWSA Federal Motor Vehicle Safety Standard, Rearview Mirrors; Federal Motor Vehicle Safety Standard, Low-Speed Vehicles. Phase-in Reporting Requirements. 49 CFR Parts 571 and 585. Docket No. NHTSA-2010-0162.

[15] City of New York (2007). Title 15, Chapter 28. Rules of the City of New York. Citywide Construction Noise Mitigation.

[16] NIOSH (2010). Safe-in-Sound Excellence in Hearing Loss Prevention Awards recognize the New York City’s noise mitigation rule [press release]. http://tinyurl.com/36c6x2u.

[17] NHTSA (2009). Incidence of Pedestrian and Bicyclist Crashes by Hybrid Electric Passenger Vehicles: Technical Report. DOT HS 811 204.

[b18-ehp.119-a30] 18The sounds used at intersections vary. For instance, Silver Spring, Maryland, uses a loud ping. Raleigh, North Carolina, uses chirping bird sounds. Barre, Vermont, uses a buzzer that Blomberg says he recently clocked at 94 dB despite the fact that the speaker was taped over, as if to try to blunt the volume, he says. But the latest technologies are more discreet, says Dave Yanchulis, an accessibility specialist with the Access Board, an independent federal agency that develops accessibility guidelines and standards under the Americans With Disabilities Act and other laws. Next spring the Access Board plans to issue draft guidelines for public rights-of-way.

[19] Robart RL, Rosenblum LD (2009). Are hybrid cars too quiet?. J Acoust Soc Am.

[20] The sounds of the Chevrolet Volt [video].

[21] Nissan LEAF forward sound [video].

[22] Electric Vehicles [website] NoiseOFF.org.

[23] Motor Vehicle Safety Act of 2010, H.R. 5381.

[24] MLIT, Japan (2010). Japanese Activities on Approaching Vehicle Audible System for HEVs and Evs.

